# A drug–drug interaction study to evaluate the impact of peficitinib on OCT1- and MATE1-mediated transport of metformin in healthy volunteers

**DOI:** 10.1007/s00228-020-02876-2

**Published:** 2020-05-16

**Authors:** Mai Shibata, Junko Toyoshima, Yuichiro Kaneko, Kazuo Oda, Tetsuya Nishimura

**Affiliations:** 1 Astellas Pharma Inc., 2-5-1 Nihonbashi-Honcho, Chuo-Ku Tokyo, 103-8411 Japan; 2 Astellas Research Institute of America LLC, Northbrook, IL USA

**Keywords:** Peficitinib, Drug–drug interaction, OCT1, MATE1, Rheumatoid arthritis

## Abstract

**Purpose:**

Peficitinib is an oral pan-Janus kinase inhibitor for the treatment of rheumatoid arthritis. Co-administration of peficitinib with metformin, a type 2 diabetes therapy, can occur in clinical practice. Hepatic and renal uptake of metformin is mediated by organic cation transporter 1 (OCT1) and OCT2, respectively, and its renal excretion by multidrug and toxin extrusion 1 (MATE1) and MATE2-K. This study investigated the effect of peficitinib on metformin pharmacokinetics in vitro and in healthy volunteers.

**Methods:**

Inhibitory effects of peficitinib and its metabolite H2 on metformin uptake into human OCT1/2- and MATE1/2-K-expressing cells were assessed in vitro. In an open-label, drug–drug interaction study, 24 healthy volunteers received a single dose of metformin 750 mg on Days 1 and 10, and a single dose of peficitinib 150 mg on Days 3 and 5–11. Blood and urine samples were collected pre-dose on Days 1 and 10, and at intervals ≤ 48 h post-dose. Metformin concentration was determined by liquid chromatography–tandem mass spectrometry and its pharmacokinetic parameters calculated.

**Results:**

Peficitinib, but not H2, inhibited metformin uptake into OCT1- and MATE1/2-K-expressing cells. Repeated-dose administration of peficitinib reduced metformin area under the concentration–time curve from 0 h extrapolated to infinity (AUC_inf_) by 17.4%, maximum plasma concentration (C_max_) by 17.0%, and renal clearance (CL_R_) by 12.9%. Co-administration of peficitinib with metformin was generally well tolerated.

**Conclusion:**

Slight changes in AUC_inf_, C_max_ and CL_R_ of metformin were observed when co-administered with peficitinib; however, these changes were considered not clinically relevant.

**Electronic supplementary material:**

The online version of this article (10.1007/s00228-020-02876-2) contains supplementary material, which is available to authorized users.

## Introduction

Rheumatoid arthritis (RA) is an autoimmune disease that is associated with chronic, painful joint inflammation [1, 2]. RA causes cartilage and bone damage [3], and in some people, progressive joint erosion is linked to physical disability and impaired quality of life [2, 4, 5]. There has been significant progress over the last 20 years in our understanding of the pathophysiology of RA, which has driven the development of effective new treatment strategies [6].

The introduction of targeted synthetic disease-modifying antirheumatic drugs (DMARDs) with novel mechanisms of action has further increased the treatment options for patients not responding sufficiently to existing DMARDs [6]. The Janus kinase (JAK) family (JAK1, JAK2, JAK3 and tyrosine kinase-2 [TYK2]) of non-receptor tyrosine kinases are implicated in the pathogenesis of RA, and are considered a promising target for RA treatment [7, 8]. A number of JAK inhibitors have been developed in recent years, with differential specificity for one or more JAKs [8]. Peficitinib (ASP015K) is an oral pan-JAK inhibitor, which has demonstrated efficacy and acceptable safety at doses up to 150 mg as once-daily therapy for moderate-to-severe RA [9–11]. Peficitinib has been approved in Japan for the treatment of RA [12]. In two Phase I, randomised, placebo-controlled trials in healthy subjects, pharmacokinetic (PK) and pharmacodynamic evaluation of single and multiple peficitinib doses showed that the drug was absorbed rapidly, and that urinary excretion accounted for 9–15% of an oral dose [13]. Three conjugated metabolites (H1, H2, H4) are produced, which show very weak in vitro pharmacological action [14]. The H2 and H4 metabolites are produced by sulphuric acid conjugation and methylation of peficitinib, respectively, and may undergo further metabolic transformation to H1 (a sulphated and methylated metabolite) [15]. Based on the assumption that peficitinib is stable in the gastrointestinal tract, a clinical mass balance study of six healthy male subjects administered with a single oral dose of ^14^C-labelled peficitinib ([^14^C]peficitinib) suggested that approximately 64% of the peficitinib dose was absorbed [15]. This was based on mean recovery of peficitinib in urine and faeces of 36.8% and 56.6%, respectively, and mean faecal excretion of 29.8% of the administered dose as unchanged peficitinib [15].

Metformin, a first-line therapy for type 2 diabetes mellitus, is among the most commonly prescribed medications in adults [16]. The liver is a major site of metformin’s pharmacological action, where it works mainly by suppressing excessive hepatic glucose production [16]. Hepatic uptake of metformin is mediated primarily by organic cation transporter (OCT)1 [16–18]. Renal uptake of metformin from the circulation into epithelial cells is facilitated primarily by OCT2, while multidrug and toxin extrusion (MATE)1 and MATE2-K, which are expressed on the apical membrane of the renal proximal tubule cell, contribute to renal excretion [16].

As the main elimination pathway of metformin is not metabolism, drug–drug interactions (DDIs) resulting from the inhibition of metformin transporters are clinically relevant [16]. Several clinical DDIs between metformin and inhibitors of OCTs and MATEs, such as cimetidine, have been reported [19]. Currently, there is no published evidence for an interaction between peficitinib or its metabolites and OCT1/2 or MATE1/2-K.

We investigated the effects of peficitinib and H2, which has the highest exposure of the three metabolites [15], on the uptake of metformin into human OCT1/2- and MATE1/2-K-expressing cells. Subsequently, we investigated the effects of peficitinib on the pharmacokinetics of metformin in a clinical DDI study in healthy subjects.

## Methods

### In vitro study

Human embryonic kidney cells (HEK293) expressing OCT1, OCT2, MATE1 or MATE2-K were incubated with [^14^C]metformin in the presence of either peficitinib or its metabolite H2 for 2 or 5 min. Cells were then washed and lysed, and the amount of radioactivity in the cell lysate was measured using liquid scintillation counting. The uptake of [^14^C]metformin into the cells in the presence of peficitinib or H2 was expressed as a percentage of the control (see online Appendix 1: In vitro study methods for full methods).

### Clinical study

#### Design and subjects

This was an open-label, single-sequence, DDI study conducted in healthy male subjects aged 20–44 years at a single centre in Japan (CPC Clinical Trial Hospital, Medipolis Medical Research Institute, Kagoshima, Japan). The primary objective was to assess the effect of multiple doses of peficitinib on the PK of a single dose of metformin.

The main inclusion criteria are as follows: age 20 to 44 years; body mass index (BMI) of ≥17.6 kg/m^2^ and < 26.4 kg/m^2^. The main exclusion criteria were: received or scheduled to receive any medications (including over-the-counter [OTC] drugs) within 7 days prior to Day 1; received peficitinib or metformin hydrochloride previously.

Subjects were admitted to hospital on Day 1 and were discharged on Day 12. As food increases peficitinib exposure [13], subjects received study drugs in the fed state (within 10 min after breakfast on the administration days) to allow investigation of the safety profile at higher peficitinib exposure. On Days 1 and 10, subjects received a single dose of metformin 750 mg (250 mg × 3 tablets). On Day 3 and Days 5–11, subjects received peficitinib 150 mg (150 mg × 1 tablet), once daily. Subjects returned for an end-of-study examination on Day 15 ± 2.

#### Concomitant medications

Concomitant use of medications and therapies, including OTC drugs, was prohibited during the study period.

#### Sample collection

Blood samples for assessment of metformin concentration in plasma were collected pre-dose and at 0.5, 1, 1.5, 2, 2.5, 3, 3.5, 4, 6, 8, 10, 12, 24, 36 and 48 h post-dose on Days 1 and 10.

Urine samples for assessment of metformin concentration were collected pre-dose (spot urine) on Days 1 and 10, and at intervals up to 48 h post-dose.

#### Pharmacokinetic assessments

Bioanalysis of metformin concentrations in plasma and urine was conducted at LSI Medience Corporation, Itabashi-ku, Tokyo (see online Appendix 1: Analysis of clinical samples for bioanalytical methods). The primary endpoints were plasma PK parameters estimated for metformin, including area under the plasma concentration–time curve from the time of dosing and extrapolated to infinity (AUC_inf_), area under the plasma concentration–time curve from the time of dosing to the last measurable concentration (AUC_last_), maximum plasma concentration (C_max_), apparent oral clearance (CL/F), time to maximum plasma concentration (t_max_) and terminal elimination half-life (t_1/2_). Urinary PK parameters estimated for metformin included the percentage of the drug dose excreted into urine from the time of dosing to the collection time of the last measurable concentration (Ae_last_%), and renal clearance (CL_R_).

#### Safety

Safety assessments were undertaken from screening through to the final study assessment. Safety was assessed by the monitoring of adverse events, vital signs (supine blood pressure, supine pulse and axillary temperature), clinical laboratory tests (haematology, biochemistry and urinalysis) and 12-lead electrocardiograms (ECGs).

Treatment-emergent adverse events (TEAEs) that occurred after Day 1 of study drug administration to immediately before Day 3 of study drug administration were those in the ‘metformin alone’ phase; those that occurred after Day 3 of study drug administration to immediately before Day 10 of study drug administration were those in the ‘peficitinib alone’ phase; and those that occurred after Day 10 of study drug administration were those in the ‘peficitinib + metformin’ phase. TEAEs were graded using the National Cancer Institute’s Common Terminology Criteria for Adverse Events (NCI CTCAE) guidelines (version 4.0) [20].

#### Statistical analysis

The planned sample size was 24 subjects, based on the precedent set by other PK studies of a similar nature [21, 22]. The safety analysis set (SAF) consisted of all subjects who received at least one dose of study drug. The PK analysis set (PKAS) consisted of subjects who received the study drug and provided at least one estimable PK parameter. Plasma PK parameters were calculated by non-compartmental analysis using Phoenix(R) WinNonlin(R) software, version 6.2 (Certara USA, Inc., Princeton, NJ, USA). Baseline demographic and other characteristics, vital signs, clinical laboratory findings, 12-lead ECG results and TEAEs were summarised for the SAF using descriptive statistics.

Statistical analyses were performed using SAS(R) software, version 9.4 (SAS Institute Inc., Cary, NC, USA). To assess the effect of peficitinib on the PK of metformin, natural log-transformed plasma C_max_, AUC_inf_, AUC_last_ and CL_R_ of metformin were used in analysis of variance (ANOVA) using the SAS MIXED procedure with treatment as a fixed effect and subject as a random effect. The least-square (LS) geometric mean ratio (GMR) and its 90% confidence interval (CI) were calculated using the exponential-transformed LS geometric mean difference and CI. Missing data were not imputed or used for analyses.

#### Ethical approval

The study protocol (ClinicalTrials.gov identifier: NCT02760342), informed consent form and information for distribution to subjects were reviewed and approved by the CPC Clinical Trial Hospital Institutional Review Board. This study was conducted in accordance with the study protocol, the International Council on Harmonization (ICH) guideline for Good Clinical Practice, applicable regulations and guidelines governing clinical study conduct, and the ethical standards as laid down in the 1964 Declaration of Helsinki and its subsequent amendments. Informed consent was obtained from all individual participants included in the study.

## Results

### In vitro study

To verify OCT1/2- and MATE1/2-K-mediated transport, the effect of typical inhibitors (quinidine for OCT1/2, cimetidine for MATE1/2-K) on the uptake of [^14^C]metformin into each transporter-expressing cell line was evaluated. Uptake of [^14^C]metformin into OCT1-, OCT2-, MATE1- and MATE2-K-expressing cells in the presence of these inhibitors was reduced to 4.7–6.9%, 10.5–12.2%, 11.9–14.7% and 8.2–15.1%, respectively, of uptake in the absence of inhibitors. These results indicated that this assay system was appropriate for assessing the inhibitory effect of peficitinib and H2 on transporter-mediated metformin uptake.

Peficitinib inhibited the uptake of [^14^C]metformin into OCT1-, OCT2-, MATE1- and MATE2-K-expressing cells, with IC_50_ values of 0.247 μmol/L, 71.4 μmol/L, 10.0 μmol/L and 20.8 μmol/L, respectively (Figs. S1–S4). Although H2 slightly inhibited [^14^C]metformin uptake, [^14^C]metformin uptake into OCT1-, OCT2-, MATE1- and MATE2-K-expressing cells at the highest concentration tested (100 μmol/L) remained 70.4%, 111.1%, 76.1% and 84.0%, respectively, of uptake in the absence of inhibitors. H2 was therefore not considered to be an inhibitor of any of the transporters tested.

### Disposition and demographics of subjects in the clinical study

A total of 24 subjects were enrolled in the study, and all received at least one dose of study drug and completed the study. All 24 subjects were included in the SAF and PKAS. Subjects were aged 23–41 years, and the mean (standard deviation [SD]) age was 32.5 (5.9) years. BMI ranged from 18.1 to 25.2 kg/m^2^, and mean (SD) BMI was 21.2 (1.9) kg/m^2^ (Table 1).

**Table 1:** Subject baseline demographics and characteristics

Parameter	Subjects in SAF (*n* = 24)
Age (years)	
Mean (SD)	32.5 (5.9)
Median	35.0
Min-max	23–41
Height (cm)	
Mean (SD)	171.9 (6.3)
Median	171.5
Min-max	159.8–184.6
Weight (kg)	
Mean (SD)	63.3 (7.7)
Median	62.2
Min-max	50.4–76.3
BMI (kg/m^2^)	
Mean (SD)	21.2 (1.9)
Median	21.3
Min-max	18.1–25.2

*BMI*, body mass index; *SAF*, safety analysis set; *SD*, standard deviation

### Effect of peficitinib on metformin pharmacokinetics

**Fig. 1:**
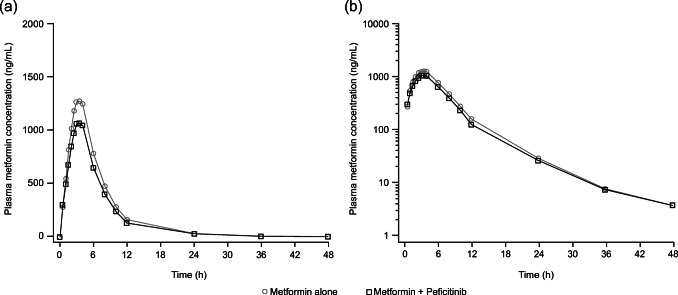
Mean plasma metformin concentrations versus time profiles by treatment (a) linear scale (b) semi-log scale (PKAS). PKAS, pharmacokinetic analysis set

Figure 1 shows the mean (SD) plasma concentration versus time profiles for metformin following administration of metformin alone or metformin co-administered with multiple doses of peficitinib. The individual profiles are shown in the Supplementary Material (Fig. S5).

With regard to exposure, for metformin co-administered with peficitinib versus metformin alone, LS GMRs (90% CI) were 0.826 (0.784–0.870) for AUC_inf_, 0.831 (0.785–0.879) for AUC_last_ and 0.830 (0.786–0.876) for C_max_ (Table 2). This showed that metformin AUC_inf_, AUC_last_ and C_max_ decreased by 17.4%, 16.9% and 17.0%, respectively, when metformin was co-administered with multiple doses of peficitinib.

**Table 2:** Plasma and urinary pharmacokinetic parameters of metformin by treatment

**Treatment/parameter**	**Plasma**						**Urine**	
	**AUC**_**inf**_**(ng∙h/mL)**	**AUC**_**last**_**(ng∙h/mL)**	**C**_**max**_**(ng/mL)**	**CL/F(L/h)**	**t**_**max**_**(h)**	**t**_**½**_**(h)**	**Ae**_**last**_**%**	**CL**_**R**_**(L/h)**
**Metformin alone**								
n	24	24	24	24	24	24	24	24
Mean (SD)	9170 (1410)	9050 (1410)	1380 (248)	83.6 (12.5)	NA	8.70 (5.11)	38.6 (3.77)	32.5 (4.37)
%CV	15.4	15.6	18.0	14.9	NA	58.8	9.8	13.5
Median	9050	8990	1380	82.9	3.00	5.48	37.7	32.6
Min-max	6820–12,500	6620–12,300	973–2030	60.2–110	2.00–4.00	3.25–19.1	33.0–49.8	22.6–41.3
**Metformin + peficitinib**								
n	23	24	24	23	24	23	24	24
Mean (SD)	7670 (1720)	7610 (1670)	1150 (242)	102 (22.4)	NA	8.63 (7.85)	28.4 (5.58)	28.6 (5.95)
%CV	22.4	22.0	21.0	21.8	NA	91.0	19.7	20.8
Median	7470	7540	1150	100	3.25	5.78	29.5	28.5
Min-max	4640–12,200	4400–11,300	809–1590	61.3–162	0.500–4.00	2.99–32.8	17.4–35.7	18.4–40.8
**Statistical assessment**								
LS GMR	0.826	0.831	0.830	–	–	–	–	0.871
90% CI of ratio	(0.784, 0.870)	(0.785, 0.879)	(0.786, 0.876)	–	–	–	–	(0.822, 0.924)

Ae_last_%, percentage of the drug dose excreted into urine from the time of dosing to the collection time of the last measurable concentration; AUC_inf_, area under the plasma concentration–time curve from the time of dosing and extrapolated to infinity; AUC_last_, area under the plasma concentration–time curve from the time of dosing to the last measurable concentration; CI, confidence interval; C_max_, maximum plasma concentration; CL/F, apparent oral clearance; CL_R,_ renal clearance; %CV, coefficient of variation presented as a percentage; LS GMR, least-square geometric mean ratio; SD, standard deviation; t_max_, time to maximum plasma concentration; t_½_, terminal elimination half-life

For metformin co-administered with peficitinib versus metformin alone, the LS GMR (90% CI) for CL_R_ was 0.871 (0.822–0.924) (Table 2). This showed that CL_R_ of metformin decreased by 12.9% when metformin was co-administered with multiple doses of peficitinib.

### Safety

There were no deaths, serious TEAEs or TEAEs leading to withdrawal of treatment reported during the study assessment period.

Overall, four (16.7%) subjects experienced a total of six TEAEs. Three (12.5%) subjects experienced a total of four mild (NCI CTCAE Grade 1) TEAEs after receiving peficitinib alone, and all four TEAEs were considered to be related to peficitinib administration. The four TEAEs were increased alanine aminotransferase (2/24, 8.3%), soft faeces (1/24, 4.2%) and increased aspartate aminotransferase (1/24, 4.2%). One (4.2%) subject experienced two mild TEAEs that were considered not to be drug related after receiving a combination of peficitinib and metformin. All TEAEs resolved without treatment.

There were no clinically significant mean changes from baseline in vital sign measurements or 12-lead ECG variables during the study.

## Discussion

Patients with RA have an increased risk of type 2 diabetes [23], and concomitant use of peficitinib and metformin represents a real-world co-administration scenario that is likely to be encountered in clinical practice. This DDI study demonstrated a PK interaction when metformin 750 mg was co-administered with multiple doses of peficitinib 150 mg. Exposure to metformin was decreased by approximately 17% and CL_R_ by 12.9%.

Prior to conducting the clinical study, we investigated the interactions of peficitinib and H2 (the metabolite of peficitinib with the highest exposure) with the human metformin transporters OCT1, OCT2, MATE1 and MATE2-K in vitro. Peficitinib inhibited OCT1-mediated metformin transport with an IC_50_ value of 0.247 μmol/L; no meaningful inhibitory effect was observed with H2. In a previous clinical study, the C_max_ of peficitinib following a single oral dose of 150 mg, under fasted conditions, was 524.5 ng/mL [24] (corresponding to 0.44 μmol/L of unbound peficitinib, assuming that 72.83% of peficitinib is protein bound [25]). Peficitinib thus has the potential to inhibit OCT1 (but not OCT2, MATE1, or MATE2-K) in clinical practice, and such inhibition would be expected to result in an increase in metformin exposure. However, in the clinical study, metformin exposure was reduced slightly when peficitinib was co-administered, suggesting that OCT1-mediated metformin transport was unaffected. This apparent lack of effect may also reflect the fact that metformin is not metabolised in the liver, where OCT1 plays a key role [16–18]. Instead, renal excretion has been reported to be the main clearance route for metformin [16]. Furthermore, other transporters, including plasma membrane monoamine transporter (PMAT), OCT3, carnitine/organic cation transporter (OCTN1) and serotonin reuptake transporter (SERT) have been implicated in metformin intestinal absorption [26]. Inhibitory effects of peficitinib on these other transporters might also have been involved in the observed decrease in metformin exposure, but any such effects are unknown as no data are available.

The in vitro findings also showed that peficitinib inhibited MATE1-mediated metformin transport with an IC_50_ value of 10.0 μmol/L, while H2 had no meaningful inhibitory effect on either MATE1 or MATE2-K. This effect of peficitinib on MATE1 might account for the slight decrease in CL_R_ of metformin observed in the clinical study. However, the concurrent reduction in metformin exposure suggests that this modest decrease in CL_R_ had a negligible impact on the other PK parameters of metformin. Our findings indicate that peficitinib would not be categorised as an OCT1 or MATE1 inhibitor, since the Japanese Ministry of Health, Labour and Welfare (MHLW) guideline on DDIs defines a weak inhibitor as a drug that changes mean exposure to a concomitantly administered medication ≥ 1.25-fold [27]. Moreover, safety data indicate that co-administration of peficitinib and metformin was well tolerated, with no serious TEAEs or TEAEs leading to treatment withdrawal during the study.

Verapamil, which may be used in type 2 diabetes mellitus patients with heart problems, is a known OCT1 inhibitor [28, 29]. Co-administration of peficitinib, verapamil and metformin may result in additive inhibitory effects on OCT1. However, it has been shown that co-administration of verapamil does not change the exposure of metformin [30]; therefore, using these three medications together may not cause further reduction in metformin exposure.

A single daily dose of peficitinib (150 mg) was used in our clinical study, which represents the standard dosage used in Japan [31] and is therefore reflective of real-world practice. One potential limitation of the study is that only male subjects aged from 20 to 44 years were enrolled. Despite this, no differences in the PK of peficitinib were observed between the sexes in previous Phase 1 studies conducted in the USA [13].

In conclusion, this study demonstrated that co-administration of peficitinib and metformin slightly reduced both exposure and renal clearance of metformin in healthy male subjects, but peficitinib 150 mg administered alone and in combination with metformin was generally well tolerated. Consequently, the interaction between peficitinib and metformin was considered not to be clinically relevant, and no dose adjustment is required when peficitinib and metformin are used concomitantly.

## Electronic supplementary material

ESM 1 (DOCX 2.43 mb)

ESM 2 (PNG 1276 kb)

ESM 3 (PNG 1276 kb)

ESM 4 (PNG 1276 kb)

ESM 5 (PNG 1276 kb)

ESM 6 (PNG 1926 kb)

ESM 7 (PNG 1926 kb)
