# Supplementary material for: A drug–drug interaction study to evaluate the impact of peficitinib on OCT1- and MATE1-mediated transport of metformin in healthy volunteers
Source: Eur J Clin Pharmacol. 2020 May 16;76(8):1135–41. doi: 10.1007/s00228-020-02876-2 (PMC7351850; doi:10.1007/s00228-020-02876-2)

# Appendix 1

## *In vitro* study methods

### Chemicals and materials

Peficitinib and H2 (both from Astellas Pharma, Inc. Tokyo, Japan) were dissolved in 50% dimethyl sulfoxide (DMSO) to give stock solutions. [^14^C]metformin was obtained from Moravek (Brea, CA, USA). Hanks’ balanced salts solution (HBSS), Dulbecco’s Modified Eagle Medium (DMEM) and fetal bovine serum (FBS) were provided by Life Technologies (Carlsbad, CA, USA). To confirm the suitability of the assay, quinidine and cimetidine, which are known inhibitors of OCT1/2 and MATE1/2-K, respectively, were obtained from Sigma-Aldrich (St. Louis, MO, USA).

### Cells

Human embryonic kidney (HEK293) cells expressing OCT1, OCT2, MATE1 and MATE2-K were established at the ADME & Tox. Research Institute (Sekisui Medical Co., Ltd, Ibaraki, Japan) by transfection of a pcDNA vector (Invitrogen, Carlsbad, CA, USA) containing full-length human transporter cDNA. Control HEK293 cells were established by transfection of the empty vector. The expression level of transporter mRNA in transfected cells was verified periodically by real-time polymerase chain reaction. The time-dependent transport of [^14^C]metformin into each transporter-expressing cell line was confirmed to be greater than that in control cells. The cells were maintained at 37^o^C with 5% CO_2_ in DMEM containing 10% FBS with antibiotic-antimycotics and L-glutamine.

### Determination of inhibitory effect on OCT1/2 and MATE1/2-K

Assays were performed in triplicate. For the OCT1, OCT2 and MATE1 transport assays, HEK293 cells expressing OCT1, OCT2 or MATE1, and control cells, were seeded in Collagen I-coated 24-well plates (BD Falcon, Franklin Lakes, NJ, USA) at a density of 2.1–2.5 × 10^5^ cells/well. For the MATE2-K transport assay, HEK293 cells were seeded in Collagen I-coated 12-well plates (BD Falcon) at a density of 3.5 × 10^5^ cells/well, incubated in a CO_2_ incubator (37°C; CO_2_, 5%) for 1 day, then transiently transfected with vectors containing human MATE2-K cDNA or empty vectors. All seeded plates were then incubated in a CO_2_ incubator (37°C; CO_2_, 5%) for 2 days.

Medium in all plates was then removed by aspiration and cells were washed with HBSS supplemented with 10 mmol/L 4-(2-hydroxyethyl)-1-piperazineethanesulfonic acid (HEPES) before preincubation with HBSS at 37°C for 15 min. After preincubation, the HBSS was replaced with test solution containing inhibitor and 10 μmol/L [^14^C]metformin, and the plate was incubated at 37°C for 2 min (OCT2) or 5 min (OCT1, MATE1, and MATE2-K). The final assay concentrations of peficitinib and H2 were 0, 1, 3, 10, 30 and 100 μmol/L, except for the assay of peficitinib with OCT1 for which the final concentrations were 0, 0.1, 0.3, 1, 3 and 10 μmol/L. Final assay concentrations of known inhibitors were as follows: quinidine: 100 μmol/L with OCT1 and 300 μmol/L with OCT2; cimetidine: 10 μmol/L with MATE1 and 100 μmol/L with MATE2-K. The final DMSO concentration was 0.2% for all assays.

After incubation, the test solution was removed, and the cells were washed once with ice-cold 0.2% bovine serum albumin in phosphate-buffered saline (PBS) and twice with 1 mL of ice-cold PBS. The PBS was removed and the cells were lysed using 0.5 mL (OCT1, OCT2, and MATE1) or 0.7 mL (MATE2-K) of 0.1 mol/L aqueous sodium hydroxide solution. After the cell lysate was mixed by pipetting, 0.3 mL (OCT1, OCT2, and MATE1) or 0.5 mL (MATE2-K) of the lysate was collected into a glass vial and mixed with 10 mL of the scintillator Hionic-fluor (PerkinElmer, Inc., Waltham, MA, USA) to measure the radioactivity using a liquid scintillation counter (2500TR [PerkinElmer, Inc.]).

Protein content was measured using the BCA Protein Assay Kit (Thermo Fisher Scientific, Inc., Waltham, MA, USA, or Pierce, Rockford, IL, USA) in accordance with the manufacture’s recommendations. The cleared volume (µL/mg protein) at each incubation time was calculated as: uptake amount (dpm/well) divided by (initial concentration [dpm/μL] ⨯ protein amount [mg/well]). Inhibitory effects, as a percentage of the control in the absence of inhibitor, were calculated from the corrected cleared volumes (cleared volume of transporter-expressing cells – cleared volume of control cells). IC_50_ values were calculated by the least squares method from the relationship between test substance concentration and metformin uptake as a percentage of the control.

## Analysis of clinical samples

The concentration of metformin in plasma and urine was measured using a validated liquid chromatography–tandem mass spectrometry (LC-MS/MS) method. The initial stage of the extraction procedure for plasma samples involved mixing each aliquot (20 µL) of the blank plasma samples with 20 µL of distilled water. An aliquot of 20 μL of internal standard (metformin-d_6_ hydrochloride; Toronto Research Chemicals, Inc., North York, ON, Canada) working solution was added to each except for the matrix blank, to which the same amount of distilled water was added. After adding 200 µL of distilled water, the samples were mixed for a few seconds, and the whole volume of each mixture loaded onto the preconditioned solid-phase Oasis^®^ WCX µElusion Plate (Waters Corporation, Milford, MA, USA). The plate was washed with 0.3 mL of methanol, and the analytes eluted with 0.2 mL of methanol/formic acid (100:2, v/v). The eluted solvent was evaporated at 40°C under a stream of nitrogen gas and the residues were reconstituted with 400 µL of reconstitution solution (acetonitrile, distilled water, ammonium formate and formic acid [850:150:0.31:1, v/v/w/v]). A 1 µL aliquot of each reconstituted sample was injected into the LC system.

The initial stage of the extraction procedure for urine samples involved mixing each aliquot (20 µL) of the blank urine samples with 200 µL of distilled water. An aliquot of 20 μL of the internal standard working solution was added to each except the matrix blank, to which the same amount of distilled water was added. The samples were diluted by adding 4 mL of distilled water and mixing for a few seconds. Each aliquot (10 µL) of the mixture was diluted by adding 400 µL of reconstitution solution (composition as above). A 1 µL aliquot of each diluted sample was injected into the LC system.

High-performance LC separation was carried out on a phosphorylcholine hydrophilic interaction liquid chromatography column (2.0 mm internal diameter × 50 mm) with a 3 μm particle size (Shiseido, Tokyo, Japan). Compounds were eluted using mobile phase composed of acetonitrile, distilled water, ammonium formate and formic acid (850:150:0.31:1, v/v/w/v) under isocratic conditions, with a flow rate of 0.8 mL/min and column temperature of 40°C. The analytical time was set at 1.5 min.

MS involved carrying out multiple reaction monitoring in the positive ion electrospray mode using an API4000 QTRAP LC-MS/MS system (AB SCIEX, Framingham, MA, USA). The first quadrupole, Q1, selected for the cationic moiety of metformin at a mass-to-charge ratio (m/z) of 130, and the internal standard (metformin-d_6_) at m/z 136. The product ions (m/z 85 for metformin and m/z 60 for the internal standard) were generated by collision-induced fragmentation within Q2 (collision gas was nitrogen, pressure setting 5 units) and detected at the electron multiplier. These product ions were chosen based on their significance within the MS/MS spectra. The ionspray voltage and temperature were maintained at 2500 V and 500°C, respectively. The ion source gas 1 (air) and 2 (air) were both set at 50 psi. The curtain gas (nitrogen) was set at 40 psi.

The standard curves for metformin in plasma and urine were linear from 5 to 2500 ng/mL and 2 to 1000 µg/mL, respectively. The validity of the analytical method was evaluated during study sample analysis by using quality control samples in each analytical run. Results for quality control samples at metformin concentrations of 10, 200 and 2000 ng/mL in plasma were accurate to within ±15% of the nominal concentration. Results for at least two-thirds of quality control samples at metformin concentrations of 4, 80 and 800 µg/mL in urine were accurate to within ±15% of the nominal concentration.

# supplementary Figures

## Figure S1. Inhibitory effect of peficitinib on the OCT1-mediated uptake of [^14^C]metformin


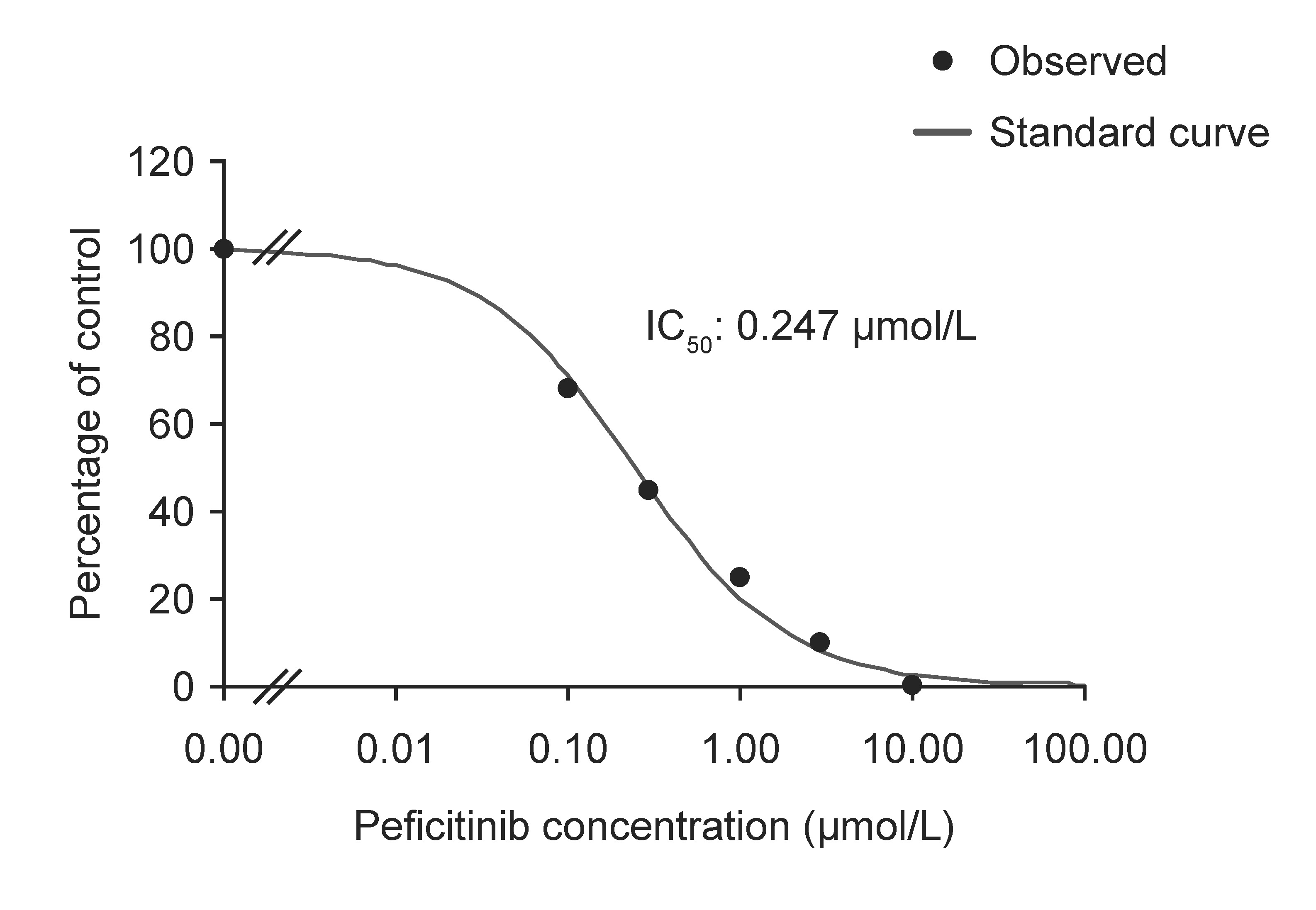


## Figure S2. Inhibitory effect of peficitinib on OCT2-mediated update of [^14^C]metformin


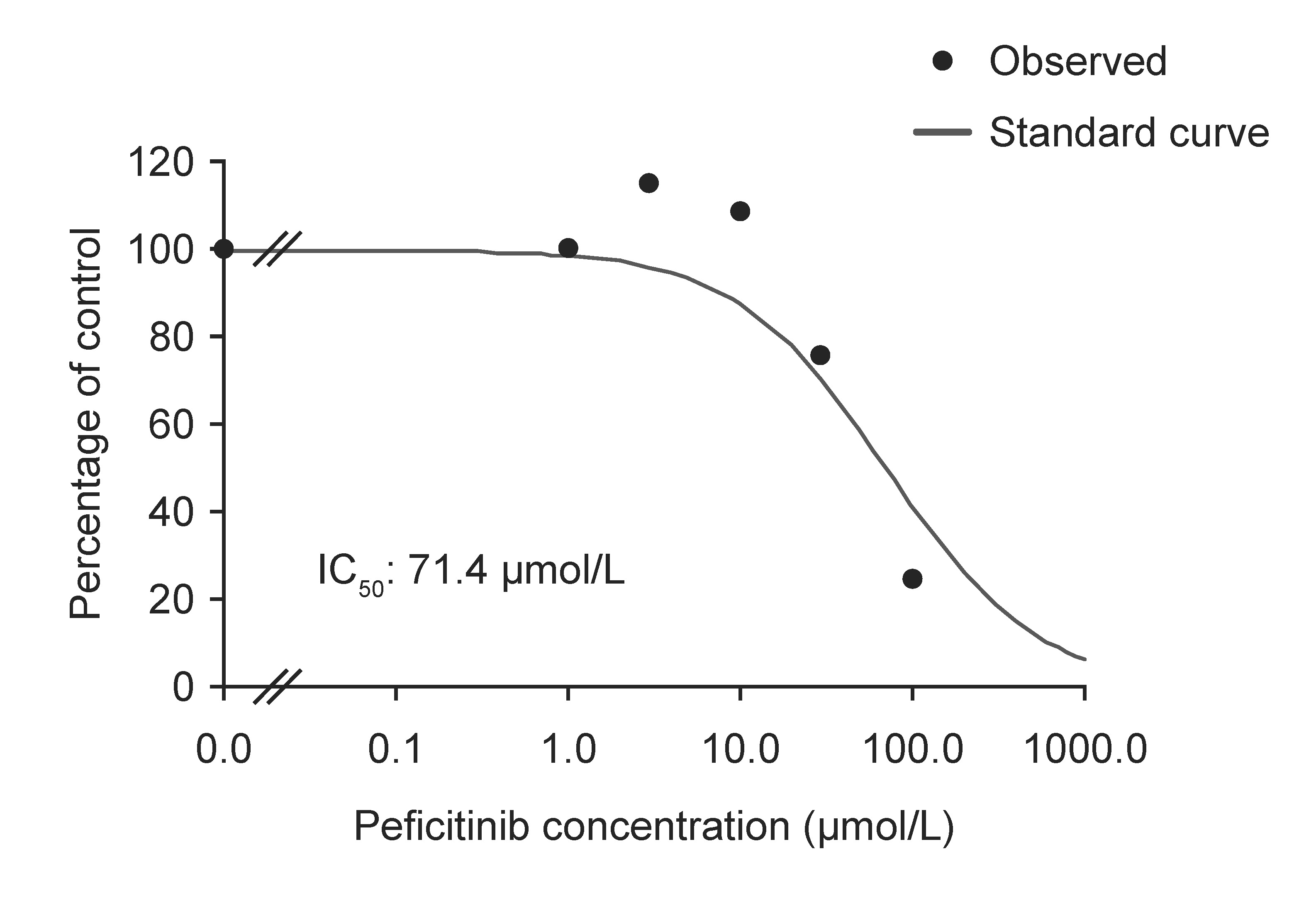


## Figure S3. Inhibitory effect of peficitinib on MATE1-mediated update of [^14^C]metformin


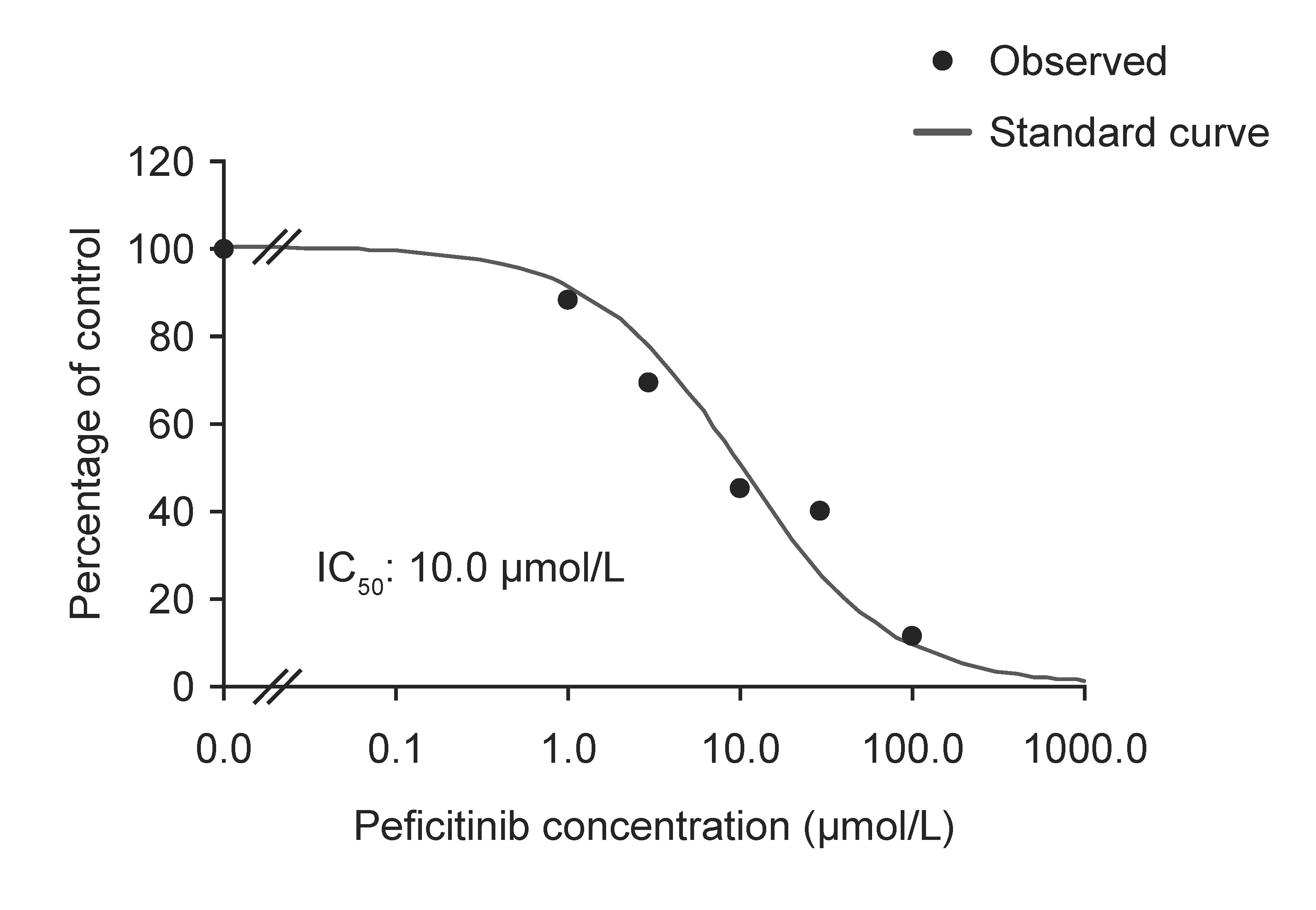


## Figure S4. Inhibitory effect of peficitinib on MATE2-K-mediated uptake of [^14^C]metformin


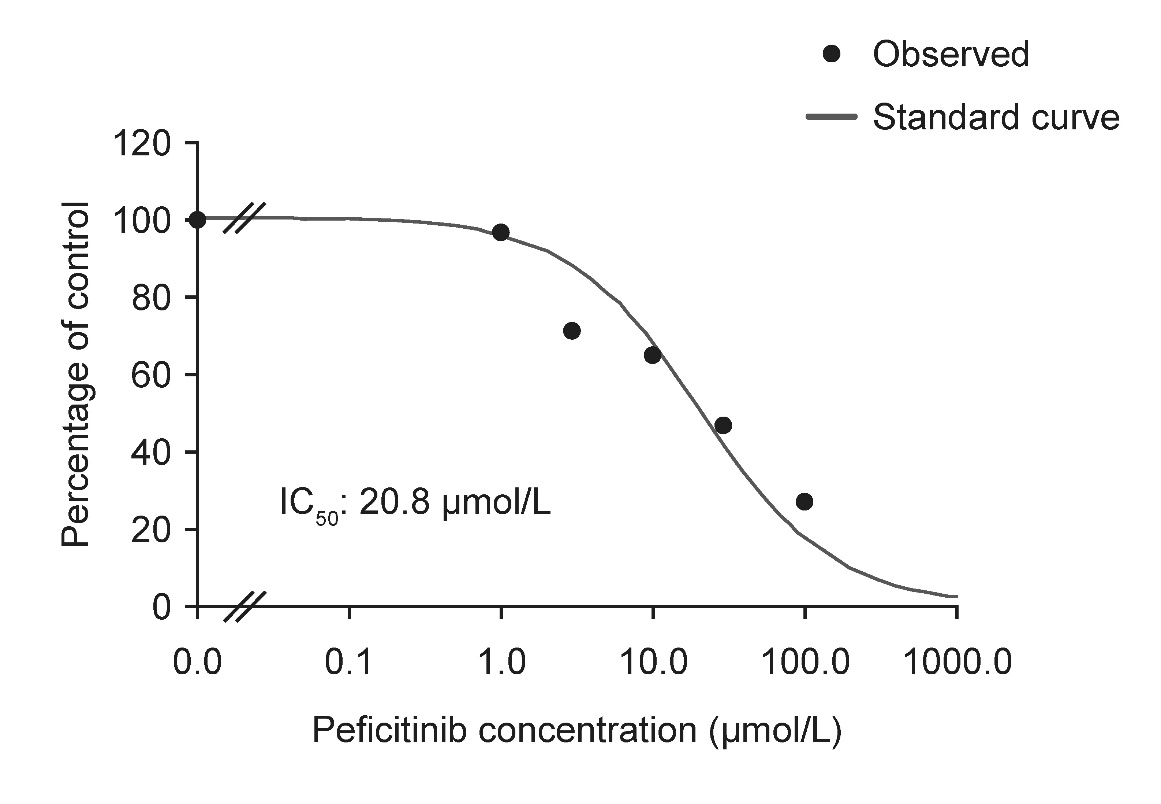


**Figure S5.** Individual plasma metformin concentrations versus time profiles by treatment, linear scale (a) metformin alone (b) metformin + peficitinib (PKAS)


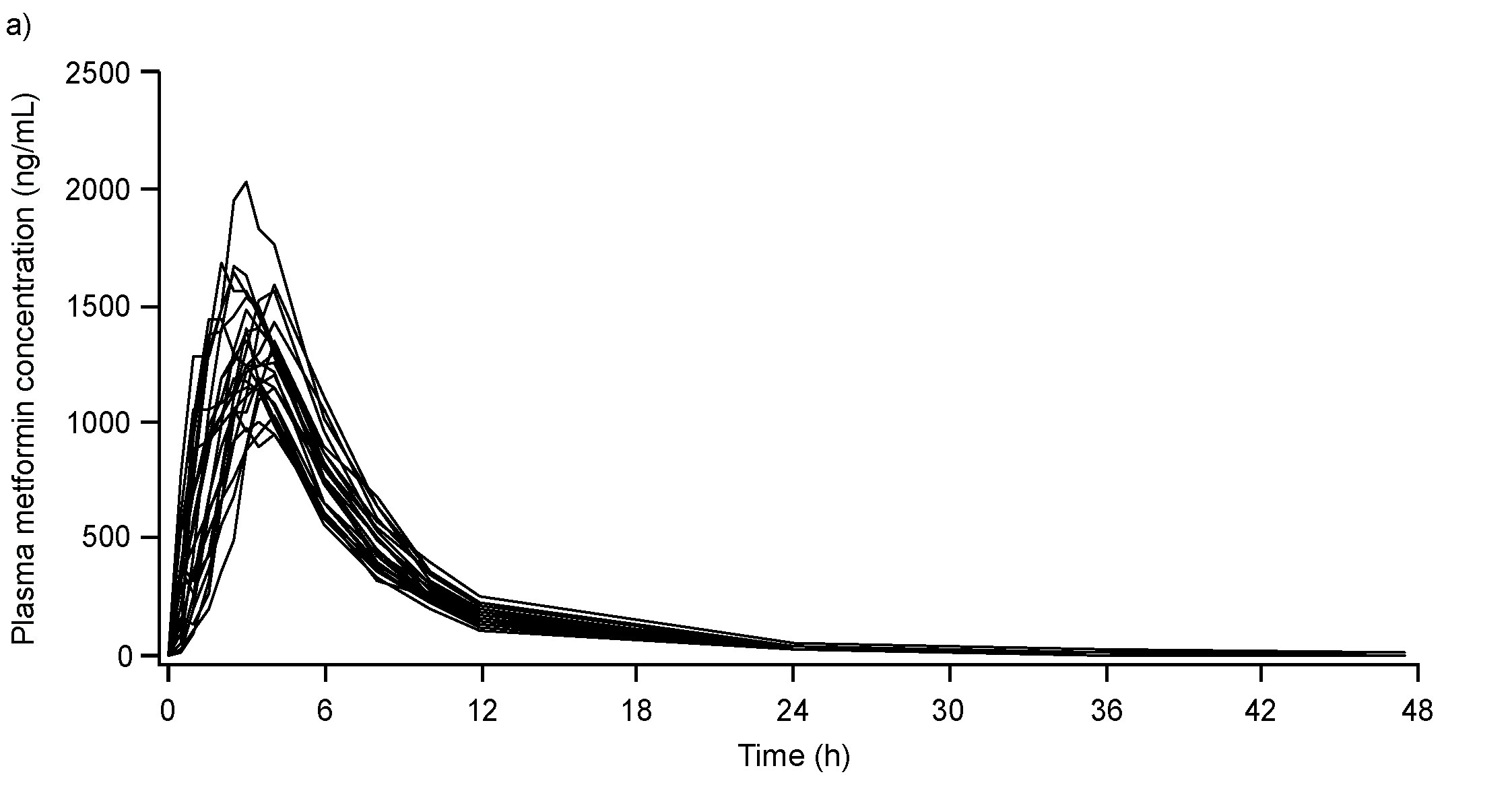

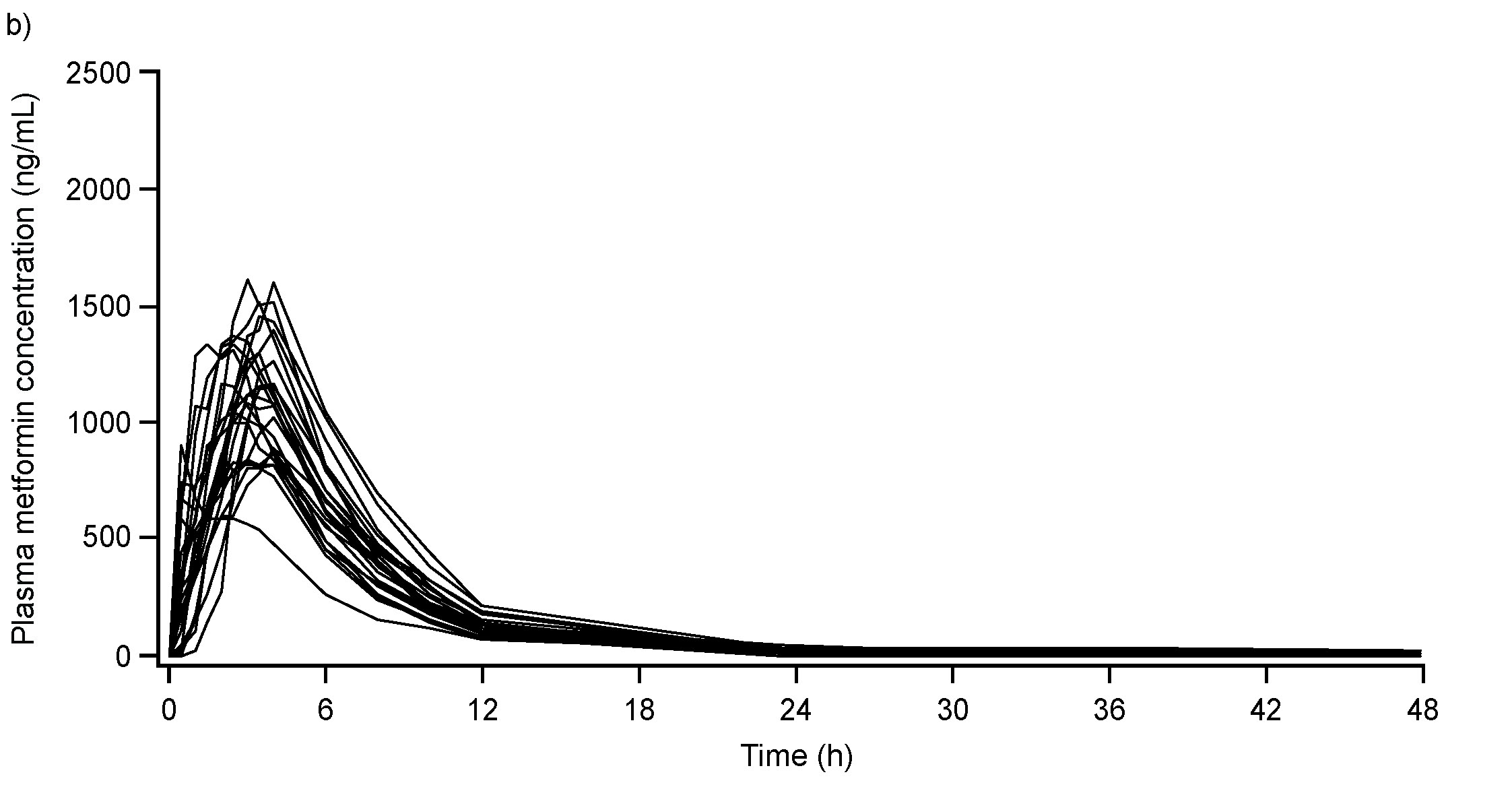

Supplement: Supplementary file 1 — (DOCX 2.43 mb) [file 228_2020_2876_MOESM1_ESM.docx]
