# Supplementary figures and images for: A drug–drug interaction study to evaluate the impact of peficitinib on OCT1- and MATE1-mediated transport of metformin in healthy volunteers
Source: Eur J Clin Pharmacol. 2020 May 16;76(8):1135–41. doi: 10.1007/s00228-020-02876-2 (PMC7351850; doi:10.1007/s00228-020-02876-2)

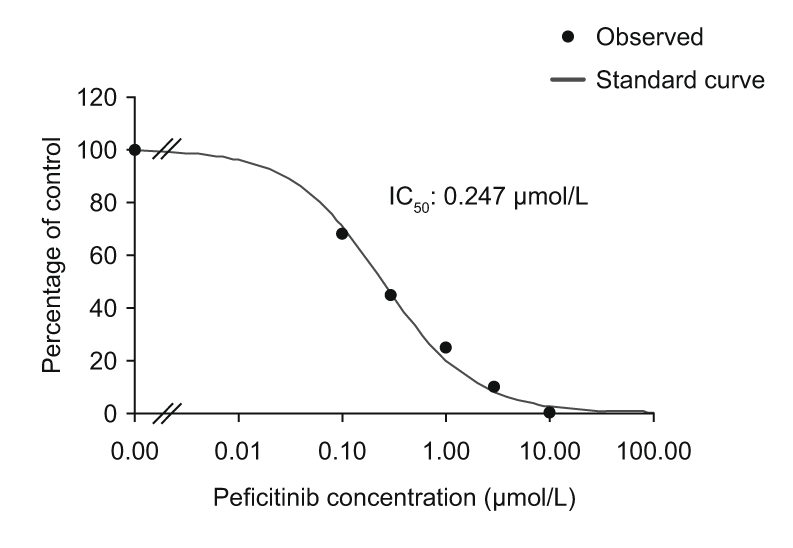

Supplement: Supplementary file 2 — (PNG 1276 kb) [file 228_2020_2876_MOESM2_ESM.png]

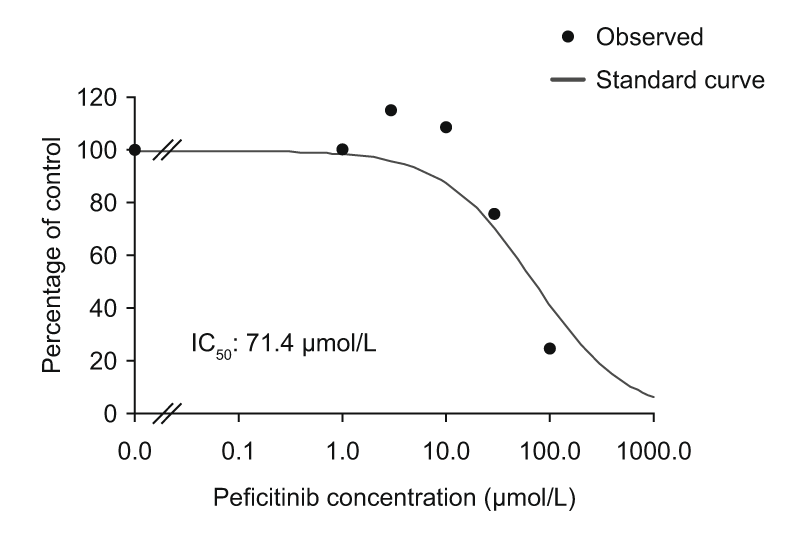

Supplement: Supplementary file 3 — (PNG 1276 kb) [file 228_2020_2876_MOESM3_ESM.png]

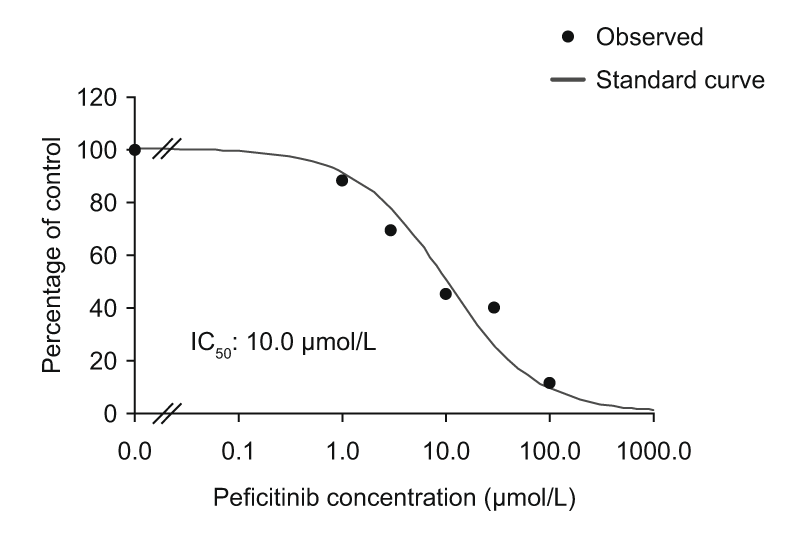

Supplement: Supplementary file 4 — (PNG 1276 kb) [file 228_2020_2876_MOESM4_ESM.png]

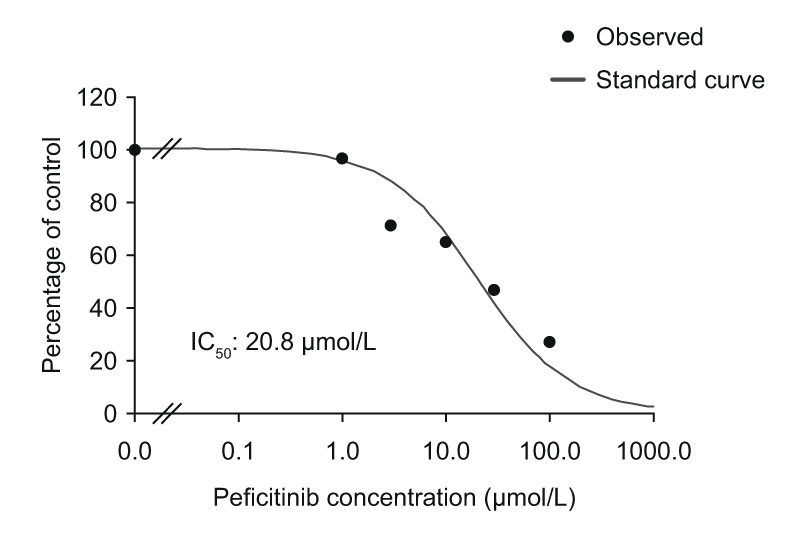

Supplement: Supplementary file 5 — (PNG 1276 kb) [file 228_2020_2876_MOESM5_ESM.png]

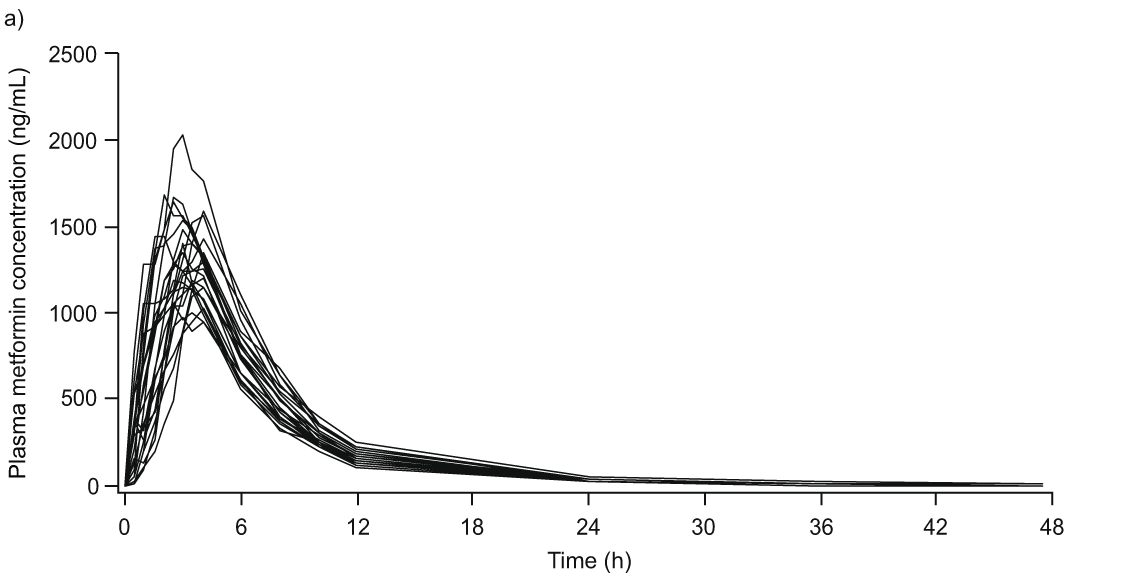

Supplement: Supplementary file 6 — (PNG 1926 kb) [file 228_2020_2876_MOESM6_ESM.png]

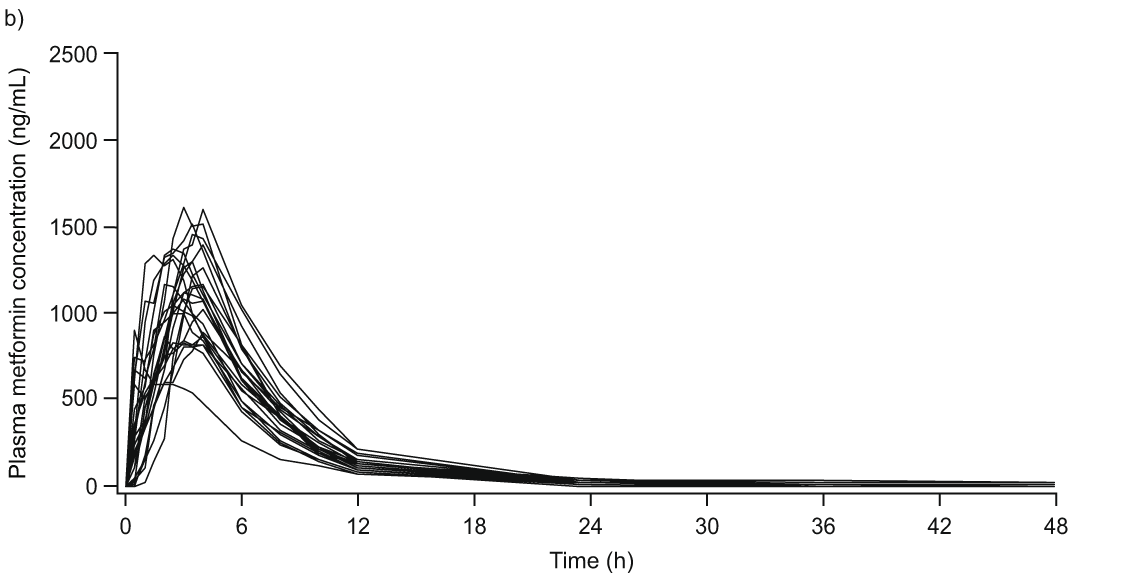

Supplement: Supplementary file 7 — (PNG 1926 kb) [file 228_2020_2876_MOESM7_ESM.png]
